# The Disproportionate Impact of COVID-19 among Undocumented Immigrants and Racial Minorities in the US

**DOI:** 10.3390/ijerph182312708

**Published:** 2021-12-02

**Authors:** Mohammad Tawhidul Hasan Bhuiyan, Irtesam Mahmud Khan, Sheikh Saifur Rahman Jony, Renee Robinson, Uyen-Sa D. T. Nguyen, David Keellings, M. Sohel Rahman, Ubydul Haque

**Affiliations:** 1 Department of Computer Science & Engineering, Bangladesh University of Engineering & Technology, West Palasi, Dhaka 1205, Bangladesh; 1505008.mthb@ugrad.cse.buet.ac.bd (M.T.H.B.); srj.buet17@gmail.com (S.S.R.J.); sohel.kcl@gmail.com (M.S.R.); 2 Department of Computer Science and Engineering, United International University, Dhaka 1212, Bangladesh; rizvi23061998@gmail.com; 3 Department of Pharmacy Practice and Administration, University of Alaska Anchorage/Idaho State University, Anchorage, AK 99508, USA; reneerobinson@isu.edu; 4 Department of Biostatistics and Epidemiology, University of North Texas Health Science Center, Fort Worth, TX 76177, USA; Uyen-sa.Nguyen@unthsc.edu; 5 Department of Geography, University of Florida, Gainesville, FL 32611, USA; djkeellings@ufl.edu

**Keywords:** unauthorized, USA, vaccine, environment, COVID-19

## Abstract

Severe acute respiratory syndrome coronavirus 2 (SARS-CoV-2), the virus responsible for coronavirus disease 2019 (COVID-19), has had an unprecedented effect, especially among under-resourced minority communities. Surveillance of those at high risk is critical for preventing and controlling the pandemic. We must better understand the relationships between COVID-19-related cases or deaths and characteristics in our most vulnerable population that put them at risk to target COVID-19 prevention and management efforts. Population characteristics strongly related to United States (US) county-level data on COVID-19 cases and deaths during all stages of the pandemic were identified from the onset of the epidemic and included county-level socio-demographic and comorbidities data, as well as daily meteorological modeled observation data from the North American Regional Reanalysis (NARR), and the NARR high spatial resolution model to assess the environment. Advanced machine learning (ML) approaches were used to identify outbreaks (geographic clusters of COVID-19) and included spatiotemporal risk factors and COVID-19 vaccination efforts, especially among vulnerable and underserved communities. COVID-19 outcomes were found to be negatively associated with the number of people vaccinated and positively associated with age, the prevalence of cardiovascular disease, diabetes, and the minority population. There was also a strong positive correlation between unauthorized immigrants and the prevalence of COVID-19 cases and deaths. Meteorological variables were also investigated, but correlations with COVID-19 were relatively weak. Our findings suggest that COVID-19 has had a disproportionate impact across the US population among vulnerable and minority communities. Findings also emphasize the importance of vaccinations and tailored public health initiatives (e.g., mask mandates, vaccination) to reduce the spread of COVID-19 and the number of COVID-19 related deaths across all populations.

## 1. Introduction

The burden of the COVID-19 pandemic, similar to other health conditions, has not been equally distributed and has been particularly detrimental to vulnerable populations, older adults, those with chronic conditions, minority populations, the poor, and those who work essential jobs [1,2]. Strong associations were found between and COVID-19 outcomes and various socio-demographic, socioeconomic, and environmental variables such as race/ethnicity and healthcare access [3,4,5,6]. Nearly 80% of hospitalized patients were over 65 years of age, and the risk of death was 23-times greater in this age group compared with those under 65 years of age [3]. Comorbidities such as cardiovascular disease (CVD), diabetes, obesity, hypertension, and cholesterol levels further increased the risk of COVID-19 fatality [7,8,9,10].

Racial and ethnic minorities in the United States (US) were disproportionately affected by COVID-19, leading to higher morbidity and mortality rates within these groups [11,12,13,14]. A Center for Disease Control (CDC) study concluded minorities in 14 states were disproportionately impacted by COVID-19 [15]. Data compiled from four different sources across seven states indicated that Black communities were disproportionately affected, and race was an independent risk factor for COVID-19 infection and mortality [1]. A study in California found that compared with than non-Hispanic white patients, non-Hispanic African American patients were 2.7 times more likely to be hospitalized, and Alaska Native people had higher death rates (10% in Alaska Native versus 6% Non-Hispanic White population), adjusting for age, sex, comorbidities, and income [11].

The COVID-19 pandemic has also exposed the increased risk of morbidity and mortality to unauthorized segments of the population [12,13,16,17,18]. Health and economic disparities among unauthorized immigrants placed them at increased risk for COVID-19 infections and deaths. The fear of deportation, healthcare access concerns, and environmental challenges (e.g., quarantine issues in multipartite households) can result in a disproportionate number of COVID-19 infections, complications, and deaths in the undocumented population [2,19]. However, COVID-19 infection rates dropped from 9.0% to 4.6% with vaccination, with over 50% reduction among individuals 65 years or older [20]. In the same period, vaccination was associated with a decrease in non-ICU hospitalizations (63.5%), ICU hospitalizations (65.6%), and deaths (69.3%) [20]. In addition to population characteristics, previous studies also suggested cold and dry environments are likely to facilitate the COVID-19 transmission [21,22,23]. For each 1 °C increase in ambient temperature and increased humidity, a decline in daily-confirmed case counts was noted for up to 14 days [24].

Taken together, surveillance of those at high risk of COVID-19 infection and/or mortality is critical for preventing and controlling the pandemic, especially with the emergence of variants and more virulent strains. We hypothesize that there are geographic clusters of COVID-19. These geographic clusters might be associated with spatiotemporal risk factors (e.g., age, sex, race, undocumented immigrants, vaccination, etc.). Advanced knowledge of outbreaks can help tailor COVID-19 mitigation efforts, especially among vulnerable and underserved communities.

## 2. Methods

Daily reported aggregated data on US county-level COVID-19 cases and deaths from the onset of the epidemic (22 January 2020) to 30 September 2021 were used to establish target variables (outcome variables) [25]. Daily meteorological modeled observation data (1 January 2020 to 30 September 2021) from the North American Regional Reanalysis (NARR), consisting of county-level temperature mean, minimum and maximum, total precipitation, and relative humidity data, and the NARR high spatial resolution model (32 km) was used to assess environmental factors (Table 1) [26] (https://psl.noaa.gov/data/gridded/data.narr.monolevel.html#source, accessed on 1 October 2021). We also used aggregated county-level meteorological features (Table 1), vaccination data, socio-demographic and comorbidities data, and the number of unauthorized people living in each county (January 2020 to 30 September 2021), as features as described in Supplementary Materials Table S1 [27,28,29].

### 2.1. Time Frames

Except for the (independent) analysis on vaccination data, we divided the whole timeline into three segments, namely, the first wave (from the onset to 30 September 2020), the second wave (1 October 2020 to 11 January 2021), and vaccination (21 January 2021 to 30 September 2021). Notably, the beginning of the second wave was assumed to be from 1 October 2020, based on expert opinions [30,31,32]. In addition to these three time segments, the entire study period is also considered as an overall time segment in our analyses.

### 2.2. Data Preprocessing

For county-level socio-demographic and comorbidities data, features that vary across populations were normalized using min–max normalization (so that the maximum value is 1 and the minimum is 0), i.e., dividing them by the total population of the respective county. For missing data, the median of the normalized value was used [33]. Meteorological data were preprocessed separately, aligning them with available COVID-19 data for the prevalence of infection and mortality. For each county, when analyzing the prevalence of COVID-19 infection and mortality, we removed data for the days’ field when the number of COVID-19 cases was zero.

Some anomalies were noted in the county-level unauthorized population data (Figure S1). The number of unauthorized populations was unavailable for some of the counties. Rather, the total number of unauthorized populations of a group of counties was available. We used the value for the aggregated unauthorized population for the groups of counties as the value for the unauthorized population for the individual counties included in that aggregated group [34]. Vaccination data and socio-demographic and comorbidities data were available at the *county* level. Only publicly available, de-identified, unlinked, and aggregated county-level data were used in this study; therefore, Investigational Review Board approval was not required.

### 2.3. Spearman Rank Order Correlation

The Spearman Rank Order Correlation coefficient is a nonparametric measurement of the relation between two variables. It measures both strength and direction of the association. It is defined as the Pearson correlation between the rank values of two variables. For *n* samples of two variables *X* and *Y*, their Spearman correlation coefficient $r_{s}$ is computed as:

(1) $$r_{s}=\rho_{rg_{X},rg_{Y}}=\frac{cov\left(rg_{X},rg_{Y}\right)}{\sigma_{rg_{X}}\sigma_{rg_{Y}}}$$

Here,

ρ denotes the Pearson correlation coefficient;

$rg_{X},rg_{Y}$ denote the rank variables corresponding to *X* and *Y*;

$cov\left(rg_{X},rg_{Y}\right)$ is the covariance of the rank variables;

$\sigma_{rg_{X}},\sigma_{rg_{Y}}$ are the standard deviations of the rank variables.

The Spearman correlation coefficient for each feature (predictor) and each outcome variable of our interest was calculated in a county-level manner as follows:

- The Spearman correlation coefficient between each *meteorological feature,* and daily COVID-19 prevalence/mortality of the counties was calculated for each time segment. In particular, we present the Spearman Correlation results of *mean*, *maximum,* and minimum temperature, total precipitation, and relative humidity against prevalence (Figure S3) and mortality (Figure S4);
- The Spearman correlation coefficient between the cumulative number of people vaccinated (per hundred) daily (predictor) and daily COVID-19 prevalence/mortality was calculated for the vaccination period;
- The Spearman correlation coefficient between socio-demographic, comorbidities features, unauthorized population and COVID-19 prevalence/mortality was calculated for each time segment;
- The Spearman correlation coefficient between each socio-demographic, comorbidities feature, and the number of people vaccinated per hundred (aggregated for the whole study period) was calculated for the vaccination period.

For all analyses, only the predictors with *p*-value < 0.05 (unadjusted) and a Spearman correlation coefficient > 0.3 were selected. We plotted the Spearman correlation coefficients on a map for analyses A and B.

### 2.4. Feature Importance

To calculate the importance of each predictor on the outcome variables, Shapley Additive Explanations (SHAP) was used [35]. Shapley Additive Explanations [36] is a method to explain individual predictions. SHAP is used to explain the prediction of an instance x by calculating the contribution of each feature to the prediction. Shapley values show how to distribute the prediction between the features fairly. A variable can be an individual feature value or a group of feature values. Any features with large absolute Shapley values are significant and used to calculate the contribution each feature makes on a single prediction.

In order to analyze the association of socio-demographic and comorbidities data with COVID-19 features, for each period (first wave, second wave, vaccination, and entire study period), we calculated the prevalence of COVID-19 infection and mortality from COVID-19 of each county. We trained several ML models, namely, Random Forest [37], XGBoost [38], and Neural Network, to predict these outcome variables from socio-demographic and comorbidities features. In our analysis, we observed that Random Forest and XGBoost fit the data better than Neural Networks in all cases. Subsequently, we selected the best model and used SHAP to explain the importance and association of each predictor with each outcome variables (Table 2).

Again, to analyze the association between people vaccinated (per hundred) and socio-demographic and comorbidities data, we considered both one dose and two doses vaccinated population as outcome variables. For both of the cases stated above, a summary plot was generated by the SHAP explainer to understand the feature contributions for each time segment using the best model in terms of Mean Squared Error (MSE) and R^2^ values (Table 2). The higher (in Y-axis) feature is in the plot, the greater is its importance. On the X-axis, we plotted SHapely values for each data point, and on the Y-axis, we plotted the features. The color of each data point indicates the importance of that feature in that particular prediction.

We attempted to identify COVID-19 hotspots in states across the United States over a 542-day timeframe from 6 April 2020 to 30 September 2021. For this purpose, we used Getis-Ord Gi * statistic, which outputs a z-score based on the attribute value and spatial weight [39,40]. Gi statistics is a well-known method to determine hot spots and cold spots in geospatial data. For more details, see the Supplementary Materials (Figure S2).

### 2.5. Codes, Environment, and Availability

We used both R (version 3.6.1, The R Foundation, Vienna, Austria) and Python (version 3.9.1, Python Software Foundation, Wilmington, DE, USA) for data analysis and model fitting. Several R packages, namely, *sf*, *sp*, *tmap*, *tmaptools*, *RColorBrewer*, etc., were used to plot maps. The R package *dplyr* was used for data analysis. Several Python packages, namely, *Pandas*, *Numpy*, *Scipy*, etc., were used for data preparation and to calculate the Spearman Rank Order Correlation. We used *SHAP, scikit-learn,* and *xgboost* packages for calculating feature importance. For plotting and generating images, the packages matplotlib and seaborn were used. All R and python codes were run on a Windows 10 machine with an Intel Core i7-8750H processor and 16 GB RAM. All data and a data dictionary were uploaded as a Supplementary Materials. All code can be found in the Github repository: https://github.com/rizvi23061998/US_DATA_ANALYSIS.git (accessed on 10 October 2021).

## 3. Results

### 3.1. Spearman Rank Order Correlation

**Figure 1:**
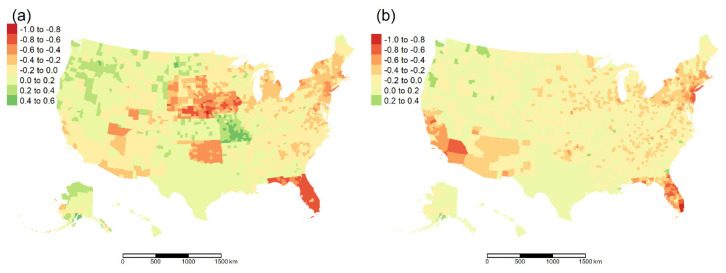
The cumulative number of people vaccinated (per hundred) daily with daily prevalence and mortality (in a county-wise manner). (**a**). Spearman correlation between daily number of cases and cumulative number of people vaccinated daily, (**b**). Spearman correlation between daily number of deaths and cumulative number of people vaccinated daily.

Spearman correlation coefficients were, for the most part, negative for the associations between cumulative daily number of people vaccinated (per hundred) and prevalence of COVID-19 infections and COVID-19 *mortality*, especially in the southern regions (Figure 1). The counties that have a higher proportion of the unauthorized population have a highly positive relationship with both COVID-19 prevalence/mortality *(*Table 3). Therefore, a high percentage of the unauthorized population is attributed to high prevalence/mortality. Table S2 of the Supplementary Materials describes the positive and negative association of each socio-demographic and comorbidities feature with prevalence and mortality.

Against the number of people vaccinated with one dose (per hundred), the Asian population has a positive correlation. As expected, population (per county) and population per square mile have a positive correlation; in contrast, *CVD* and Diabetes have a negative correlation (Table 4). It is to be noted that people vaccinated (per hundred) with two doses show almost identical correlation values. That is why only the analysis on the vaccinated population with one dose is provided.

**Figure 2:**
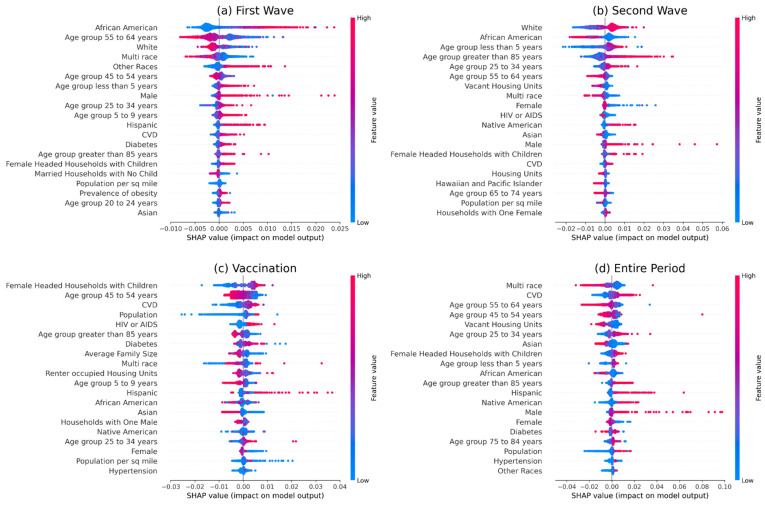
We used SHAP to understand the impact of different features on the prevalence of COVID-19 and described the results in this figure. Shapely values were calculated for each socio-demographic and comorbidities-related feature. A summary was plotted for each time segment. In the figure, indices (**a**–**d**) indicate plots for different time segments: (**a**) first wave, (**b**) second wave, (**c**) vaccination period, and (**d**) the entire study period. In the plot, a higher position implies greater importance for a feature (the actual values in the Y-axis do not have any significance).

**Figure 3:**
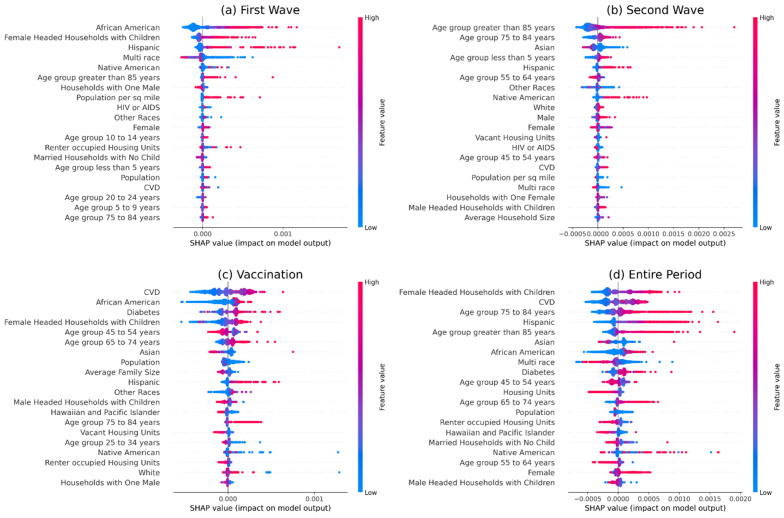
We used SHAP to understand the impact of different features on mortality and described the results in this figure. Shapely values were calculated for each socio-demographic and comorbidities-related feature. A summary was plotted for each time segment. In the figure, indices (**a**–**d**) indicate plots for different time segments: (**a**) first wave, (**b**) second wave, (**c**) vaccination period, and (**d**) the entire study period. In the plot, a higher position implies greater importance for a feature.

Our analysis on socio-demographic and comorbidities features reveals different behavior in different time segments when prevalence is used as the outcome variable. During the first wave, we see a positive association with the African American population and a negative association with the White population (Figure 2, Supplementary Materials Table S3). The relation flips during the second wave and subsequently restores for the African American *population* in the vaccination period. Next, the prevalence of *CVD* has a positive relation (most impactful feature over the entire study period). The age group of 45 to 64 years mostly shows a negative association, and the age group of ≥85 years has a positive association before the vaccination period and negative afterward. The age group of <5 years and 25 to 34 show positive associations. The multiracial population shows a negative association (second most impactful feature over the entire study period). Hispanic and Native American populations mostly have a positive association. In contrast, the Asian population has a negative association except during the first wave (Figure 2). Mostly different results are observed when mortality was used as the outcome variable. A female-headed household with children has a significantly positive association (most impactful feature over the entire study period). African American and Hispanic populations have a positive association, whereas the *Asian* population has a negative association. Except for the first wave, *CVD* and the prevalence of diabetes were found to have a positive association. Both mortality and *prevalence* show a similar association with the age groups. The older population also seems to have a positive correlation. The feature importance of the age group of ≥85 years decreased significantly during the vaccination period. The age group 45 to 54 years has negative relation (Figure 3).

### 3.2. Analysis of Vaccination Data

**Figure 4:**
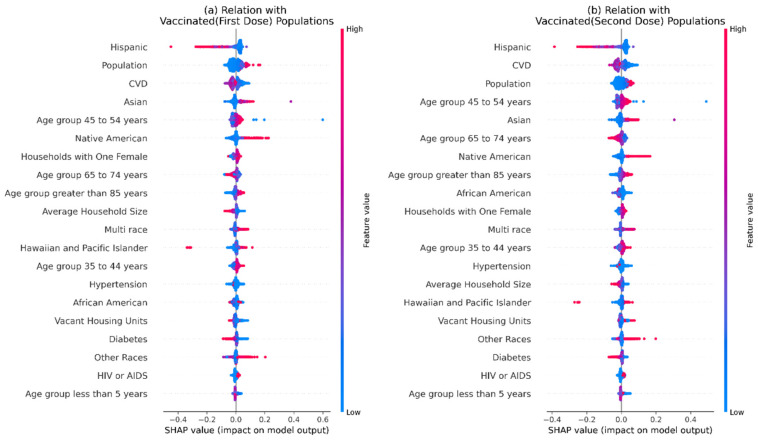
We used SHAP to understand the impact of different features on the number of people vaccinated (per hundred) and described the results in this figure. Shapely values were calculated for each socio-demographic and comorbidities-related feature. A summary is plotted here. Here, a higher position implies greater importance for a feature. We used both (**a**) vaccinated population with one dose and (**b**) vaccinated population with two doses as outcome variables.

From our analysis of vaccination data against socio-demographic and comorbidities features, we observed almost identical results for a vaccinated population with one and two doses. The high importance of different races is noticed. Hispanic population shows a negative association, whereas Asian, Native American, Multi-racial, and Hawaiian and Pacific Islander population show positive association. Comorbidities such as *CVD* and Diabetes have a negative association. The age group of 35 to 54 and the age group of ≥85 show positive association, but, interestingly, the age group 65 to 74 shows a negative association (Figure 4).

From our hotspot analysis, we found that the spread of COVID-19 first started at the upper east coast. Then by 14 May 2020, the whole east coast was exposed and predicted as a hotspot, and moved west gradually. Until 6 June 2020, the whole West Coast was a cold spot, i.e., there were very little to no cases of COVID-19 until 23 June 2020, the hotspot moved from the east coast to the lower west coast through Texas and other lower states. By 19 July 2021, the whole West Coast was predicted as a hotspot, while the whole East Coast was predicted as a cold spot. The hotspot was located on the West coast and lower parts until 9 October 2020, and then, the hotspot moved to the center and again to the east coast. By 14 November 2020, the whole country was a hotspot. Then the hotspot moved again to the lower west coast by 1 January 2021, while most of the East Coast was cold spots. By 17 February 2021, the hotspot moved to the lower right part of the US. Then, the hotspot was located again on the entire east coast by 2 March 2021, when the West Coast was a cold spot. By 7 June 2021, the hotspot moved to the lower part of the US, and by 20 June 2021, it moved to the West Coast and fluctuated from the lower part to West Coast until the end (30 September 2021). See Figure S2 for the choropleth diagram of hotspots in the US.

## 4. Discussion

Our results are consistent with other published studies [16,41,42,43,44]. COVID-19 prevalence and mortality are significantly high in counties living a high proportion of the unauthorized population and minority communities, in communities with a high prevalence of *CVD* and diabetes, and among elders [4,12,13,19,45]. Even before the pandemic, access to healthcare facilities was typically low among undocumented immigrants [41]. Barriers such as cost, lack of knowledge of resources, and fear of deportation are among those noted [14]. Extending government assistance is important, and perhaps creating a low-cost “insurance plan” for uninsured individuals regardless of citizenship status would be beneficial if barriers are removed [42]. Further mistrusts on the public-healthcare-for-all concept, with no exception to unauthorized populations, will negate the effectiveness of all identified resolutions. It is crucial to overcome language barriers, resource scarcity, and predatory job sites to seek immediate care for any potential symptoms or even preventive services, and should not tie with the negative impact on future adjustment to their immigration status [16]. Educating communities about available resources in a culturally appropriate manner (through community health workers who speak the same language, can relate to cultural beliefs, etc.) is important to promoting healthcare utilization, reduce risk, provide much-needed service to immigrants, and ensure all Americans are safe [46]. Creating programs that reach out to vulnerable undocumented communities can help to build trust with these communities and relay information that can help to curb the spread of COVID-19.

The findings of this study are also consistent with previous findings that show the infection rate is higher in counties where ethnic minorities such as Black, Hispanic, Alaska Natives, and Hawaiian/Pacific Islanders are higher compared with the White population [1]. A positive association with prevalence (except for the second wave) was seen higher in counties where the Black community has a high population, whereas in the counties where the White community has a higher proportion of population showed an exact inverse relationship. A more rigorous surveillance system, vaccination coverage, promotion of education to fight against vaccine hesitancy, and mask mandates may help curb the spread of infection among minority communities [47,48,49].

Our study, similar to other published studies, suggests counties that have a high proportion of an older population (≥85 years of age group) had a higher prevalence of COVID-19 and higher mortality throughout the four time segments [50,51,52]. However, it is important to note that counties generally composed of older adults were more likely to be vaccinated. Our findings suggest that as the number of vaccinations increases, the prevalence and mortality decrease in the county; however, no direct association was observed between climatic parameters, COVID-19 prevalence, and mortality. However, in counties where more people had a greater number of comorbidities, in particular *CVD* and/or diabetes (Supplementary Materials Table S1), individuals were at greater risk of morbidity and mortality [3,7,8,9]. Vaccinations were shown to decrease risk in this vulnerable population; therefore, steps need to be taken to increase vaccinations to prevent mortality and morbidity.

Lastly, as expected, the correlation between mean, minimum, and maximum temperature and COVID-19 *prevalence* follows the same pattern (Figure S3). From our hotspot analysis, it seemed that COVID-19 started to spread from the East Coast and gradually spread to the lower part, and then went to the West Coast. It fluctuated from the East Coast to the West Coast only through the lower part (Texas, NM, USA), but to our surprise, it rarely traveled northward, possibly due to low traffic and rural environment limiting spread.

Relative humidity showed a positive correlation on the eastern and western regions during vaccination, a negative correlation on western and northern middle regions when the whole period is considered, and little to no correlation during other time segments.

There might have some reporting bias, such as misreporting and lack of reporting by various states and counties. This study used aggregated county-level data. The observed associations between COVID-19 prevalence and mortality and the risk factors were ecologic and not at the level of individuals [53,54]. Our findings suggest broad relationships between county-level population characteristics and the impacts of COVID-19, but these associations are likely confounded by many variables not captured by this study, and a causal or individual-level interpretation should not be made.

## 5. Conclusions

This study applies several ML models and spatiotemporal approaches to support rapid detection of geographic clusters of COVID-19 outbreaks that may focus on COVID-19 mitigation and management strategies on a county level. Strong correlations were found between both COVID-19 cases and deaths with the number of people vaccinated, the prevalence of CVD, and diabetes, age, and minority population. There was also a strong relationship between the number of unauthorized immigrants and the prevalence of COVID-19 cases and deaths. These findings confirm that already vulnerable populations were disproportionately impacted by COVID-19 and that public health initiatives need to focus on targeting and tailoring initiatives that promote health equity, and the fundamental changes are required on a system level.

## Figures and Tables

**Table 1:** Description of meteorological features.

Feature	Description
Mean Temperature	The average temperature of the day in degrees Celsius
Min Temperature	Minimum temperature of the day in degree Celsius
Max Temperature	Maximum temperature of the day in degree Celsius
Total Precipitation	Total precipitation of the day in millimeter
Relative Humidity	Relative humidity of the day in percentage

**Table 2:** Best models for Feature Importance calculations.

Outcome Variable	Time Segment	Best Model
COVID-19 prevalence	First wave	Random Forest with 500 estimators
	Second wave	Random Forest with 500 estimators
	Vaccination	XGBoost with 300 estimators
	The entire study period	XGBoost with 300 estimators
COVID-19 mortality	First wave	Random Forest with 500 estimators
	Second wave	Random Forest with 300 estimators
	Vaccination	XGBoost with 150 estimators
	The entire study period	XGBoost with 180 estimators
People vaccinated with one dose (per hundred)	Vaccination	XGBoost with 700 estimators
People vaccinated with two doses (per hundred)		XGBoost with 550 estimators

**Table 3:** Spearman coefficient of prevalence and mortality against the unauthorized population.

Feature (County Level)	Time Segment	Spearman Correlation Coefficient
COVID-19 prevalence	First wave	0.83
	Second wave	0.75
	Vaccination	0.72
	The entire study period	0.77
COVID-19 mortality	First wave	0.74
	Second wave	0.68
	Vaccination	0.71
	The entire study period	0.75

All *p*-values are <0.01.

**Table 4:** Spearman correlation between each predictor and people vaccinated with one dose per hundred (only predictors with spearman coefficient > 0.3 with a *p*-value < 0.05 were selected).

Predictor	Spearman Coefficient
Asian	0.42
Population	0.40
CVD	−0.35
Population per sq. mile	0.31
Diabetes	−0.30

All *p*-values are <0.01.

## Data Availability

All data were uploaded as Supplementary Materials, and codes were posted in the Github repository.

## Supplementary Materials

The following are available online at https://www.mdpi.com/article/10.3390/ijerph182312708/s1, Table S1: Description of socio-demographic and comorbidities features (Source: https://www.arcgis.com/home/item.html?id=8e5c3c6e1fa94e379553e199dcc4e777&view=list#data accessed on 1 October 2021). Table S2: Association of socio-demographic and comorbidities features with COVID-19 prevalence/mortality (Only features with a spearman coefficient of >0.3 with a 5% significance level were selected). Table S3: Supplementary Materials: Impact of socio-demographic and comorbidities features on prevalence/mortality (only features with high importance were chosen). Figure S1: Spatial distribution of unauthorized population in the US. Figure S2: Geographic hotspots of COVID-19 in the USA. Figure S3: Spearman correlation values were calculated for each meteorological feature with the daily prevalence (at a county-level). We conducted this for each time segment and plotted the values of each county on the map. In the figure, indices (a–d) indicate plots for different time segments: (a) first wave, (b) second wave, (c) vaccination period, and (d) the entire study period. Figure S4: Spearman correlation values were calculated for each *meteorological feature* with the *daily mortality* (in a county-wise manner). We conducted this for each time segment and plotted the values of each county on the map. In the figure, indices (a–d) indicate plots for different time segments: (a) first wave, (b) second wave, (c) vaccination period, and (d) the entire study period.
