# Supplementary material for: The Disproportionate Impact of COVID-19 among Undocumented Immigrants and Racial Minorities in the US
Source: Int J Environ Res Public Health. 2021 Dec 2;18(23):12708. doi: 10.3390/ijerph182312708 (PMC8656825; doi:10.3390/ijerph182312708)
Supplement: Supplementary file 1 [file ijerph-18-12708-s001.zip › ijerph-1393026 supplementary.pdf]

International Journal of
Environmental Research and Public Health

MDPI

Supplementary Material

Table S1. Description of socio-demographic and comorbidities features (Source: https://www.arcgis.com/home/item.html?id=8e5c3c6e1fa94e379553e199dcc4e777&view=list#data (accessed on 1 October 2021).

Feature	Description
Population per sq. mile	Population density (in 2013) (no. of people per square mile)
White	Number of white people
African American	Number of African American people
Native American	Number of Native American people
Asian	Number of people of Asian race
Hawaiian and Pacific Islander	Number of Hawaiian and Pacific Islanders
Hispanic	Number of Hispanic people
Other races	Aggregated number of people of other races
Multi-racial	Number of people belonging to multiple races (those who identify with two or more races)
Male	Number of males
Female	Number of females
Age group < 5 years	Number of people below age 5
Age group 5 to 9 years	Number of people aged between 5 and 9
Age group 10 to 14 years	Number of people aged between 10 and 14
Age group 15 to 19 years	Number of people aged between 15 and 19
Age group 20 to 24 years	Number of people aged between 20 and 24
Age group 25 to 34 years	Number of people aged between 25 and 34
Age group 35 to 44 years	Number of people aged between 35 and 44
Age group 45 to 54 years	Number of people aged between 45 and 54
Age group 55 to 64 years	Number of people aged between 55 and 64
Age group 65 to 74 years	Number of people aged between 65 and 74
Age group 75 to 84 years	Number of people aged between 55 and 84
Age group ≥ 85 years	Number of people aged 85 and above
Households	Number of households
Average Household Size	The average number of people per household
Households with One Male	Number of 1-person male households
Households with One Female	Number of 1-person female households
Married Households with Children	Number of married-couple households with children
Married Households with No Child	Number of married-couple households with no child
Male Headed Households with Children	Number of male-headed households with children
Female Headed Households with Children	Number of female-headed households with children
Families	Number of families
Average Family Size	The average number of people per family
Housing Units	Number of total housing units
Vacant Housing Units	Number of vacant housing units
Owner-occupied Housing Units	Number of owner-occupied housing units
Renter-occupied Housing Units	Number of renter-occupied housing units
Prevalence of Obesity	Number of people with obesity
Hypertension	Number of people suffering from hypertension
Diabetes	Number of people affected with diabetes
CVD	Number of people suffering from Cardiovascular disease
HIV/AIDS	Number of people HIV/AIDS positive

Table S2. Association of socio-demographic and comorbidities features with COVID-19 prevalence/mortality (Only features with a spearman coefficient of > 0.3 with *p*-value < 0.05 were selected).

**Time Segment**	**COVID-19 prevalence**		**COVID-19 mortality**	
	**Positive spearman**	**Negative spearman**	**Positive spearman**	**Negative spearman**
First wave	• African American<br>• Female-Headed Households with Children<br>• Age group 25 to 34 years<br>• Age group < 5 years<br>• Age group 20 to 24 years<br>• Average Household Size	• White<br>• Married Households with No Child<br>• Age group 55 to 64 years<br>• Owner-occupied Housing Units<br>• Age group 45 to 54 years<br>• Housing Units	• African American<br>• Female-Headed Households with Children	• White
Second wave	• White<br>• Age group ≥ 85 years	• African American	• Age group 75 to 84 years<br>• Age group ≥ 85 years<br>• Male<br>• Owner-occupied Housing Units<br>• White<br>• Married Households with No Child	• Population per sq. mile<br>• Asian<br>• Population<br>• Average Family Size<br>• Average Household Size
Vaccination	• Female-Headed Households with Children<br>• African American		• CVD<br>• Diabetes<br>• Female-Headed Households with Children	
The entire study period	• Age group < 5 years		• CVD<br>• Female-Headed Households with Children<br>• Female<br>• Diabetes	• Asian

Table S3. Impact of socio-demographic & comorbidities features on Prevalence/Mortality (only features having high importance were chosen).

Time Segment	COVID-19 prevalence		COVID-19 mortality	
	Positive impact	Negative impact	Positive impact	Negative impact
First wave	• African American<br>• Other races<br>• CVD<br>• Age group < 5 years<br>• Age group ≥ 85 years<br>• Hispanic<br>• HIV/AIDS<br>• Age group 5 to 9 years<br>• Age group 25 to 34 years<br>• Male<br>• Diabetes<br>• Female-Headed Households with Children	• Age group 55 to 64 years<br>• White<br>• Multi-racial<br>• Age group 45 to 54 years	• African American<br>• Female-Headed Households with Children<br>• Hispanic<br>• Native American<br>• Age group ≥ 85 years<br>• Population per sq. mile<br>• HIV/AIDS<br>• Other races<br>• Female<br>• Age group 10 to 14 years<br>• Renter-occupied Housing Units<br>• Age group < 5 years<br>• Population	• Multi-racial<br>• Households with One Male<br>• Married Households with No Child
Second wave	• Age group ≥ 85 years<br>• White<br>• Age group < 5 years<br>• Age group 25 to 34 years<br>• Native American<br>• Female-Headed Households with Children<br>• Male<br>• CVD	• African American<br>• Vacant Housing Units<br>• Multi-racial<br>• Age group 55 to 64 years<br>• Female<br>• Asian<br>• Age group 65 to 74 years	• Age group ≥ 85 years<br>• Age group 75 to 84 years<br>• Age group < 5 years<br>• Hispanic<br>• Native American<br>• White<br>• Male<br>• CVD	• Asian<br>• Age group 55 to 64 years<br>• Other races<br>• Female<br>• Vacant Housing Units<br>• Age group 45 to 54 years<br>• Population per sq. mile<br>• Multi-racial
Vaccination	• Female-Headed Households with Children<br>• CVD<br>• Population<br>• HIV/AIDS<br>• Diabetes<br>• African American<br>• Multi-racial<br>• Hispanic<br>• Age group 65 to 74 years	• Age group 45 to 54 years<br>• Age group ≥ 85 years<br>• Renter-occupied Housing Units<br>• Age group 5 to 9 years<br>• Households with One Male<br>• Average Family Size<br>• Female	• CVD<br>• African American<br>• Diabetes<br>• Female-Headed Households with Children<br>• Age group 65 to 74 years<br>• Hispanic<br>• Other races<br>• Age group 75 to 84 years	• Age group 45 to 54 years<br>• Asian<br>• Population<br>• Average Family Size<br>• Male Headed Households with Children<br>• Hawaiian and Pacific Islander<br>• Vacant Housing Units<br>• Age group 25 to 34 years

The entire study period	• CVD<br>• Age group 25 to 34 years<br>• Female-Headed Households with Children<br>• Age group < 5 years<br>• Age group ≥ 85 years<br>• Hispanic<br>• Diabetes<br>• Population<br>• Native American	• Multi-racial<br>• Age group 55 to 64 years<br>• Age group 45 to 54 years<br>• Vacant Housing Units<br>• Asian<br>• African American<br>• Female	• Female-Headed Households with Children<br>• CVD<br>• Age group 75 to 84 years<br>• Hispanic<br>• Age group ≥ 85 years<br>• African American<br>• Diabetes<br>• Age group 65 to 74 years	• Asian<br>• Multi-racial<br>• Age group 45 to 54 years<br>• Housing units<br>• Renter-occupied Housing Units<br>• Hawaiian and Pacific Islander<br>• Married Households with No Child

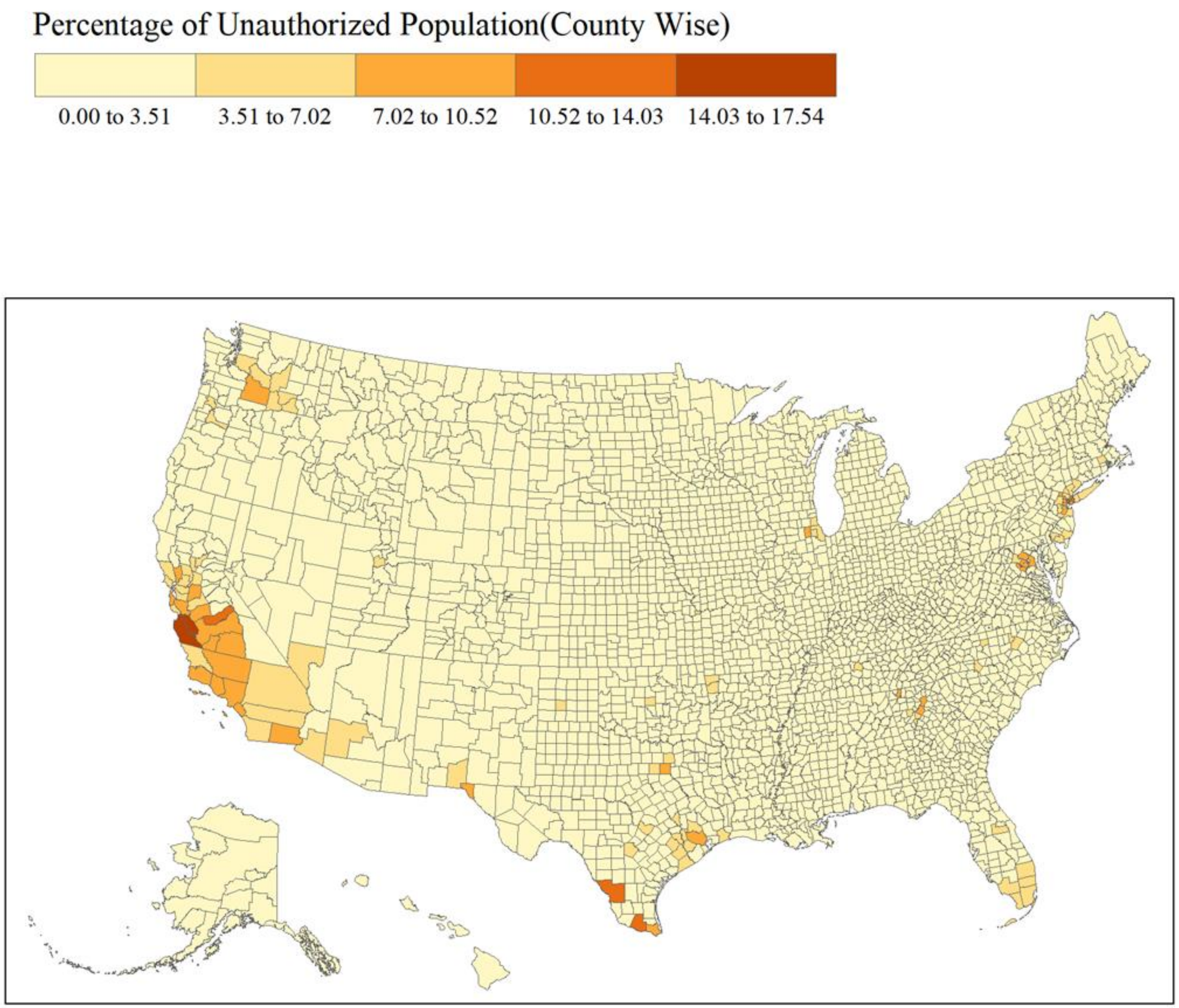

Figure S1. Spatial distribution of unauthorized population in the US.

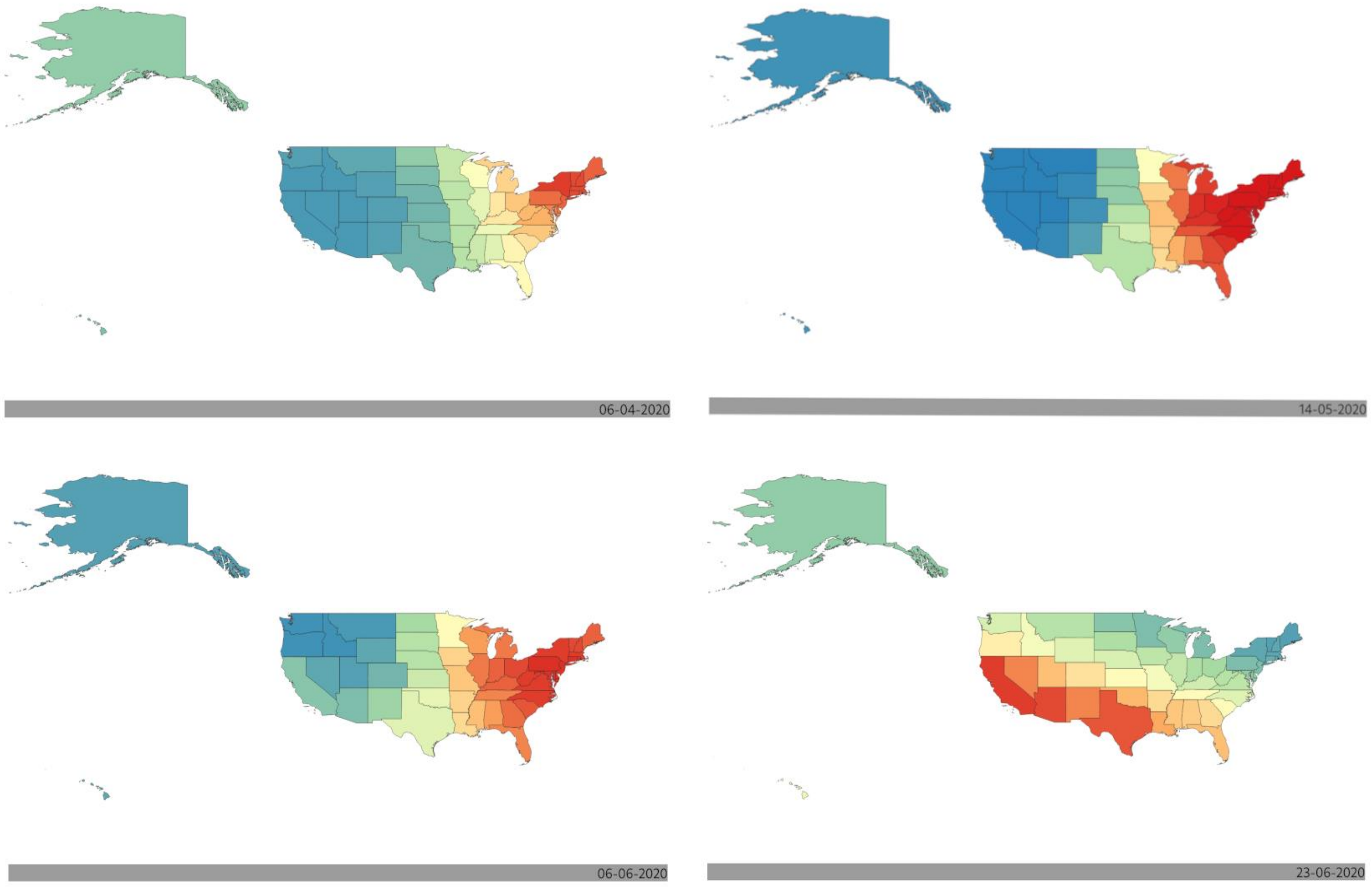
06-04-2020
14-05-2020
06-06-2020
23-06-2020

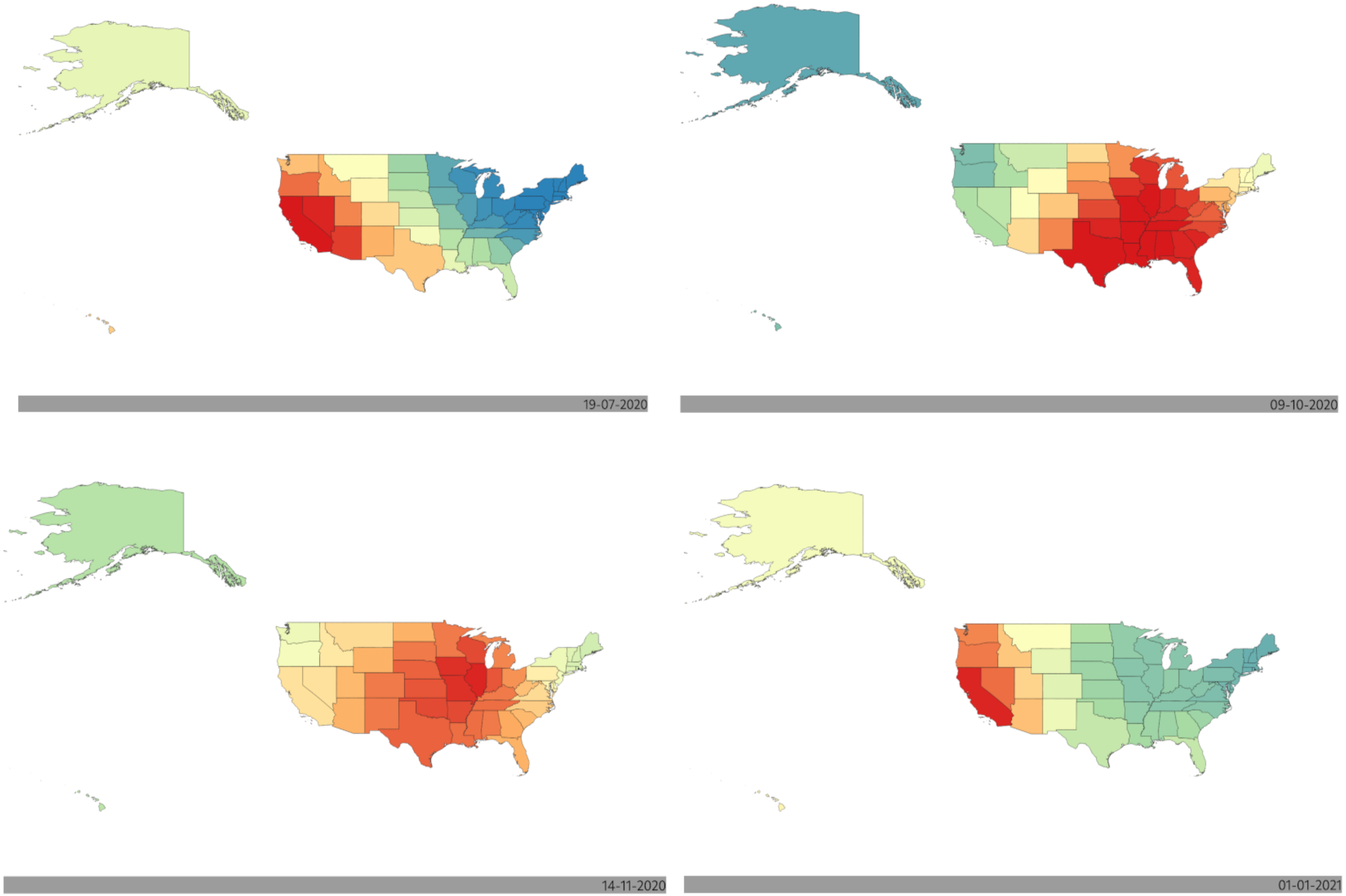
19-07-2020
09-10-2020
14-11-2020
01-01-2021

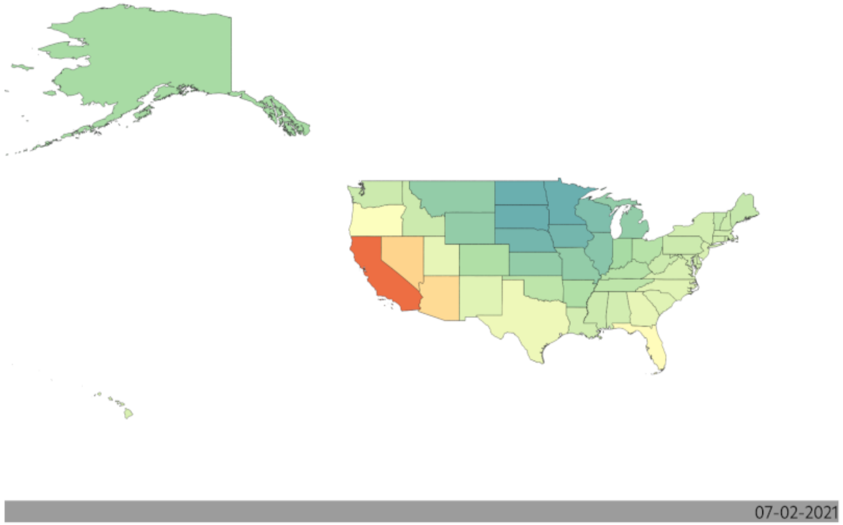
07-02-2021

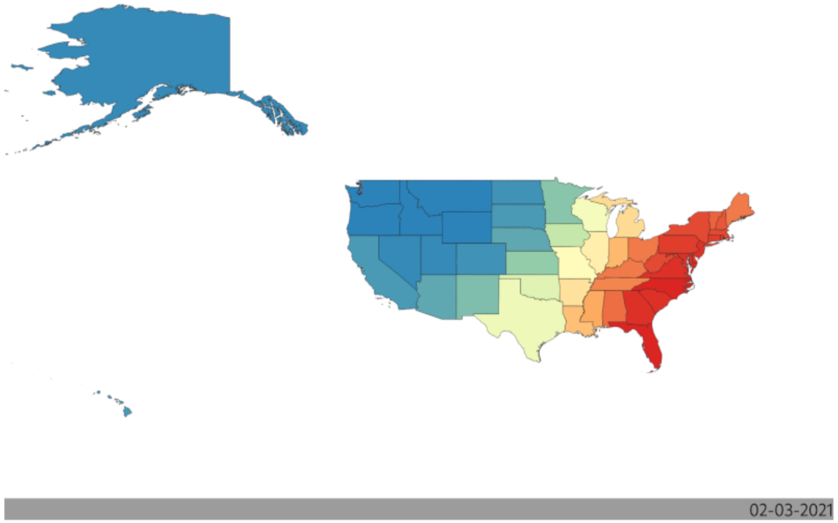
02-03-2021

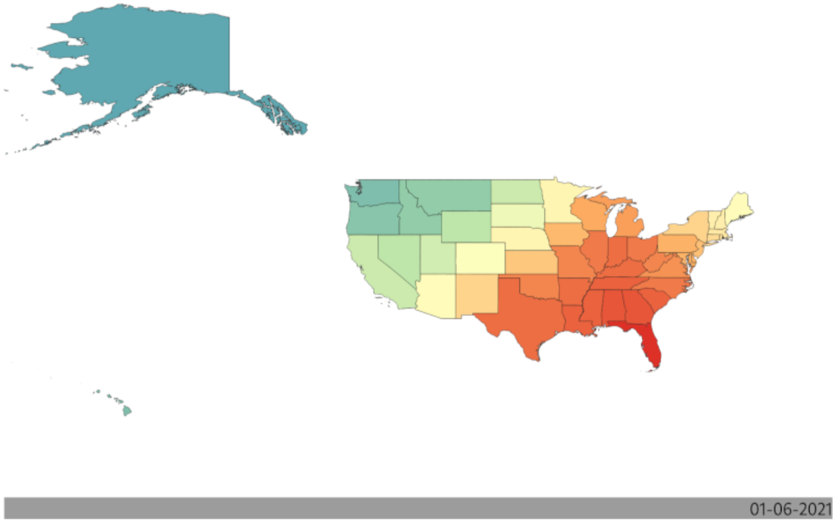
01-06-2021

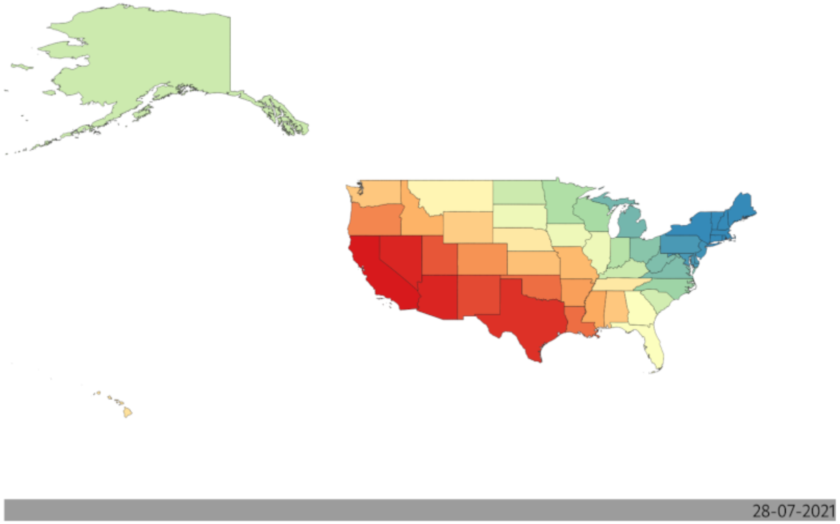
28-07-2021

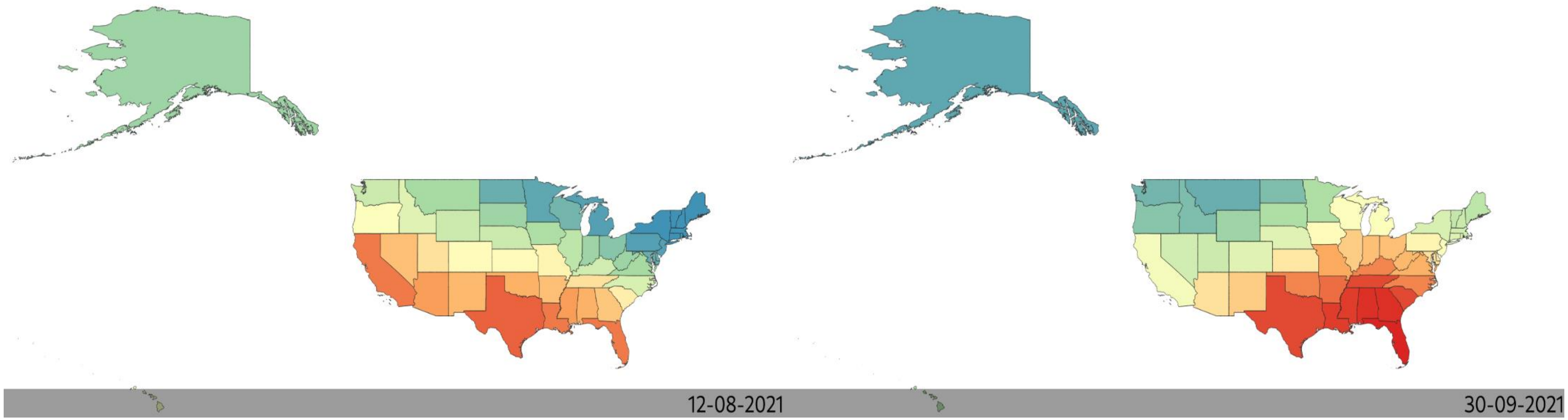

Figure S2. Geographic hotspots of COVID-19 in the USA.

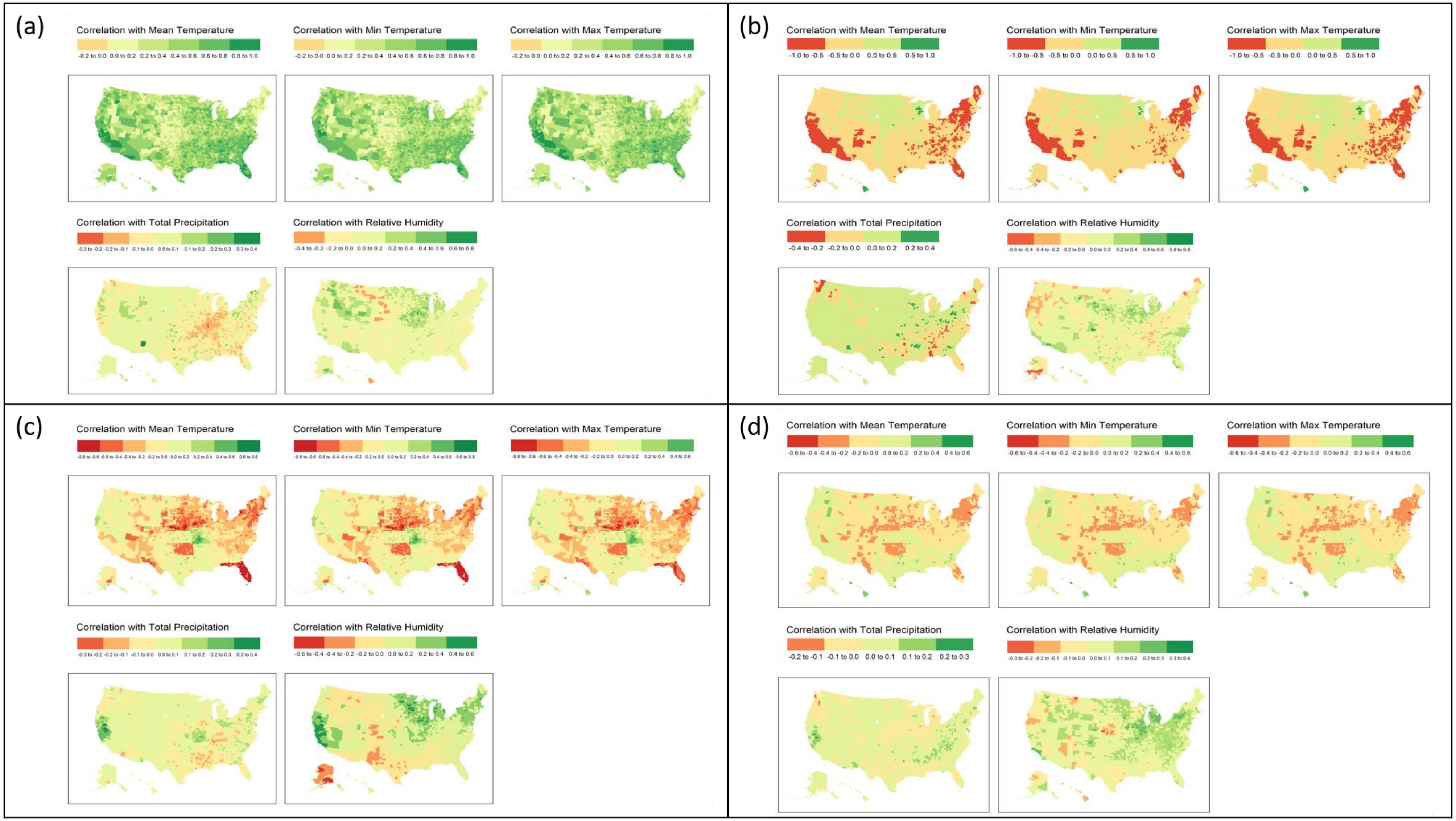

Figure S3. Spearman correlation values were calculated for each meteorological feature with the daily prevalence (at a county-level). We conducted this for each time segment and plotted the values of each county on the map. In the figure, indices **(a)–(d)** indicate plots for different time segments: (a) first wave, b) second wave, c) vaccination period, and d) the entire study period).

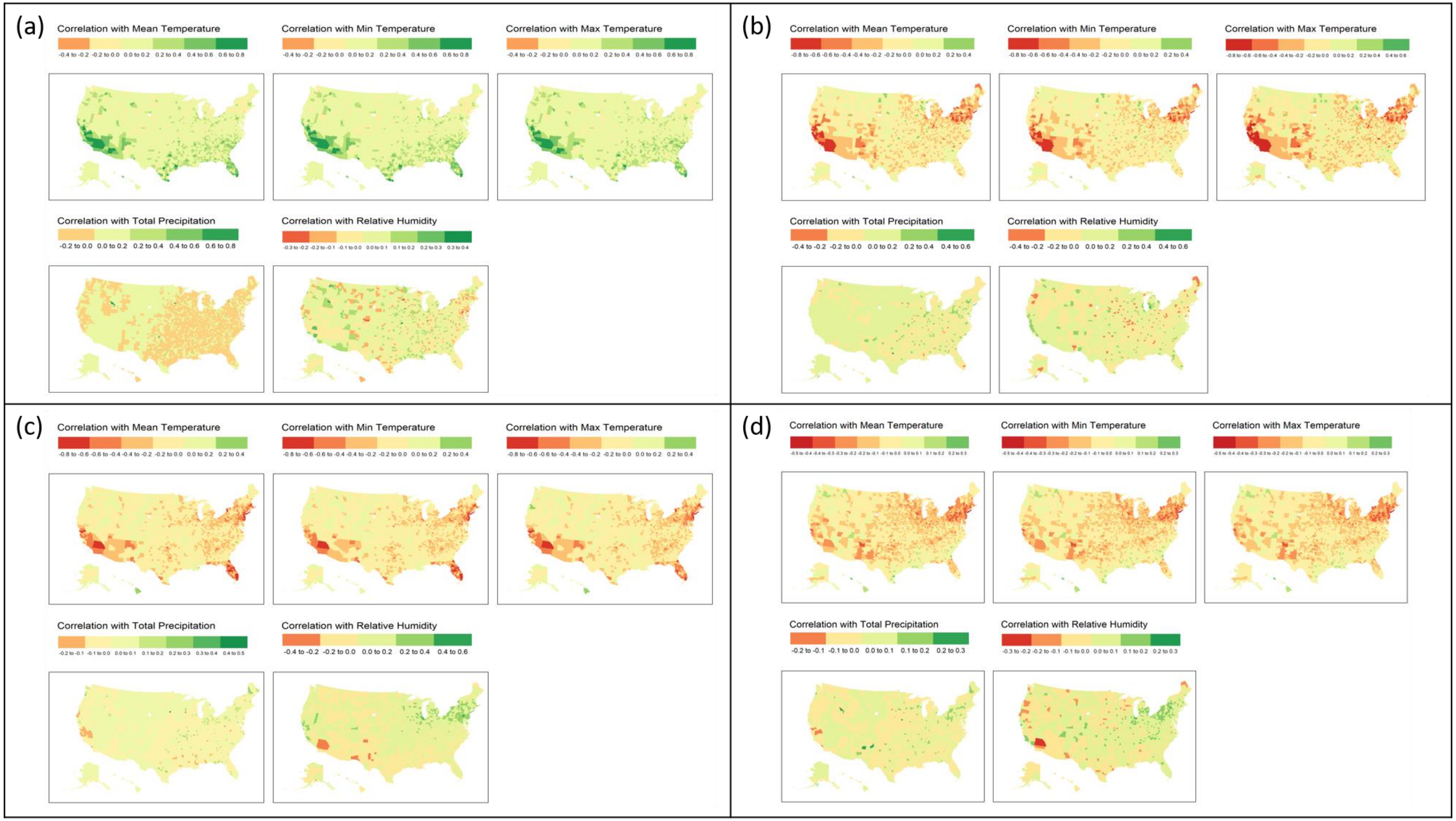

Figure S4. Spearman correlation values were calculated for each meteorological feature with the daily mortality (in a county-wise manner). We conducted this for each time segment and plotted the values of each county on the map. In the figure, indices (**a**)–(**d**) indicate plots for different time segments: (a) first wave, b) second wave, c) vaccination period, and d) the entire study period).
